# Supplementary figures and images for: Protective effects of Colla Corii Asini Collagen Peptides on D-galactose injection combined with UVB irradiation-induced aging in mice
Source: PLoS One. 2025 Feb 13;20(2):e0317302. doi: 10.1371/journal.pone.0317302 (PMC11825052; doi:10.1371/journal.pone.0317302)

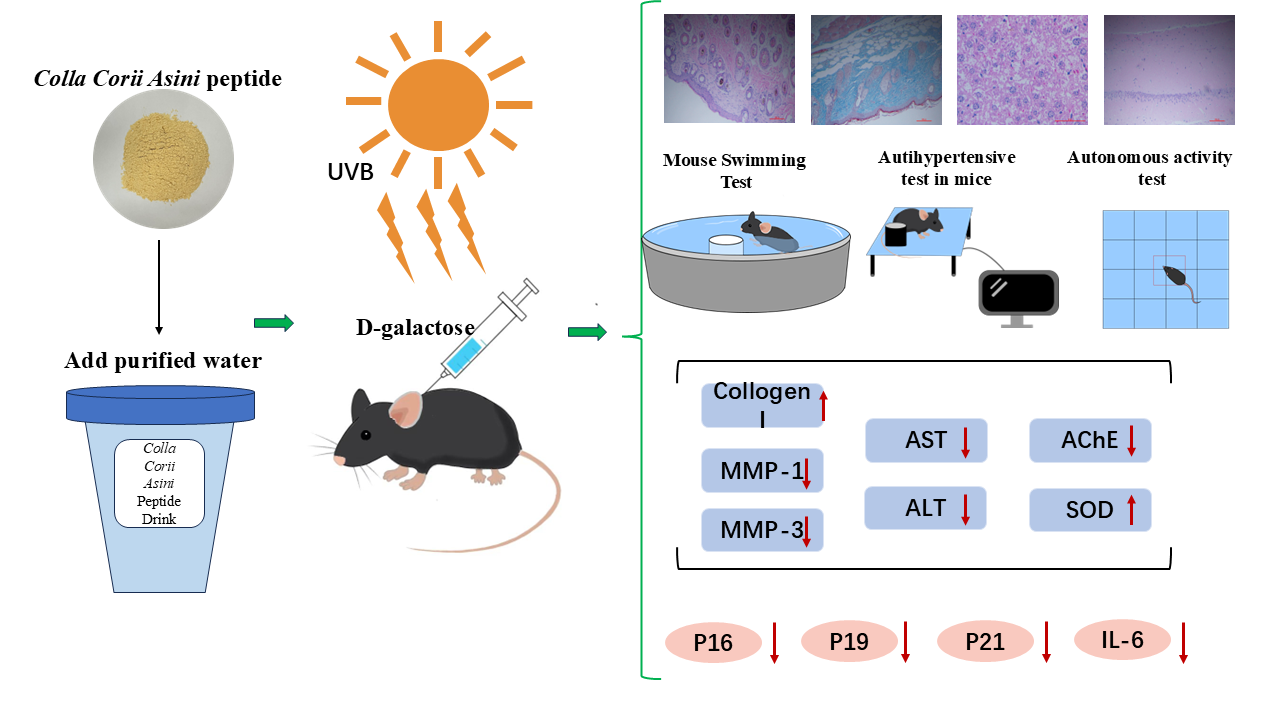

Supplement: S1 Graphical abstract — (TIF) [file pone.0317302.s001.tif]
